# Immune Repertoire Diversity Correlated with Mortality in Avian Influenza A (H7N9) Virus Infected Patients

**DOI:** 10.1038/srep33843

**Published:** 2016-09-27

**Authors:** Dongni Hou, Tianlei Ying, Lili Wang, Cuicui Chen, Shuihua Lu, Qin Wang, Eric Seeley, Jianqing Xu, Xiuhong Xi, Tao Li, Jie Liu, Xinjun Tang, Zhiyong Zhang, Jian Zhou, Chunxue Bai, Chunlin Wang, Miranda Byrne-Steele, Jieming Qu, Jian Han, Yuanlin Song

**Affiliations:** 1 Department of Pulmonary Medicine, Zhongshan Hospital, Fudan University, Shanghai, 200032, China; 2 Key Laboratory of Medical Molecular Virology of Ministries of Education and Health, School of Basic Medical Sciences, Fudan University, Shanghai, 200032, China; 3 Department of Respiratory Medicine, Shanghai Public Health Clinical Center, Fudan University, Shanghai, 201508, China; 4 Division of Pulmonary and Critical Care Medicine, Department of Medicine, University of California, San Francisco, CA 94143, USA; 5 HudsonAlpha Institute for Biotechnology, Alabama, AL35806, USA; 6 Department of Pulmonary Medicine, Ruijin Hospital, Shanghai Jiaotong University, Shanghai, 200025, China; 7 Department of Pulmonary Medicine, Zhongshan Hospital, Qingpu Branch, Shanghai, 200032, China

## Abstract

Specific changes in immune repertoires at genetic level responding to the lethal H7N9 virus are still poorly understood. We performed deep sequencing on the T and B cells from patients recently infected with H7N9 to explore the correlation between clinical outcomes and immune repertoire alterations. T and B cell repertoires display highly dynamic yet distinct clonotype alterations. During infection, T cell beta chain repertoire continues to contract while the diversity of immunoglobulin heavy chain repertoire recovers. Patient recovery is correlated to the diversity of T cell and B cell repertoires in different ways – higher B cell diversity and lower T cell diversity are found in survivors. The sequences clonally related to known antibodies with binding affinity to H7 hemagglutinin could be identified from survivors. These findings suggest that utilizing deep sequencing may improve prognostication during influenza infection and could help in development of antibody discovery methodologies for the treatment of virus infection.

Influenza A (H7N9) is an emerging virus of avian origin that has caused three waves of infections since February 2013. As of February 2015, a total of 571 laboratory-confirmed cases have been reported to WHO, including 212 deaths^1^. New cases were notified in 2016 from China. H7N9 infection induced lethal complications like severe pneumonia and acute respiratory distress syndrome, and currently no specific treatment is available for this highly contagious viral infection^2^,^3^,^4^.

Effective humoral and cellular immune responses in influenza infection are critical for patient recovery from H7N9 infection^5^,^6^,^7^,^8^. Quantitative and qualitative analysis of antiviral immunity may aid in understanding the state of immune system and guide the therapeutics. Immune repertoire analysis based on next-generation sequencing (NGS) is a novel approach to analyze alterations during the antiviral immune response^9^,^10^,^11^. Many studies have reported the overwhelmingly diverse and dynamic property of immune repertoire changes in response to the antigen stimuli such as vaccination or allergy^12^,^13^,^14^. However, the changes of immune repertoire in clinical infections caused by lethal pathogens and their influence on patient recovery remain unknown.

In this study we explored alterations in the human immune repertoires after H7N9 infection. Specifically, we compared the dynamic behavior of T cell and B cell repertoires and analyzed signatures of these highly convergent immune repertoires. Also we identified antibody sequences from these NGS data. These results provide direct insights into the immune response, especially distinct features of T cell and B cell repertoire behaviors, after human influenza A (H7N9) infection and suggest potential implications in antibody development and prognostication.

## Results

### Next-generation sequencing results

Our study utilized peripheral blood samples collected at multiple time points from patients infected with H7N9 virus in 2013. Among these patients, 10 of 15 (66.7%) recovered from infection. From a total of 35 samples, deep sequencing based on Illimina MiSeq platform produced 150 base pairs surrounding the CDR3 of each T cell receptor β chain (TRB), and 250 base pairs covering CDR1 ~ 3 and beginning of C region of each Ig chain. Sequencing depth was comparable among samples (Supplementary Table S3). Because of the limited quantity of extracted RNA from blood samples, no reads of IGL or IGK were obtained from a non-survivor (patient J) and a survivor (patient C). These two samples were excluded from all analysis.

### Ig heavy chain repertoire showed highly dynamic changes over time in a survived individual

**Figure 1:**
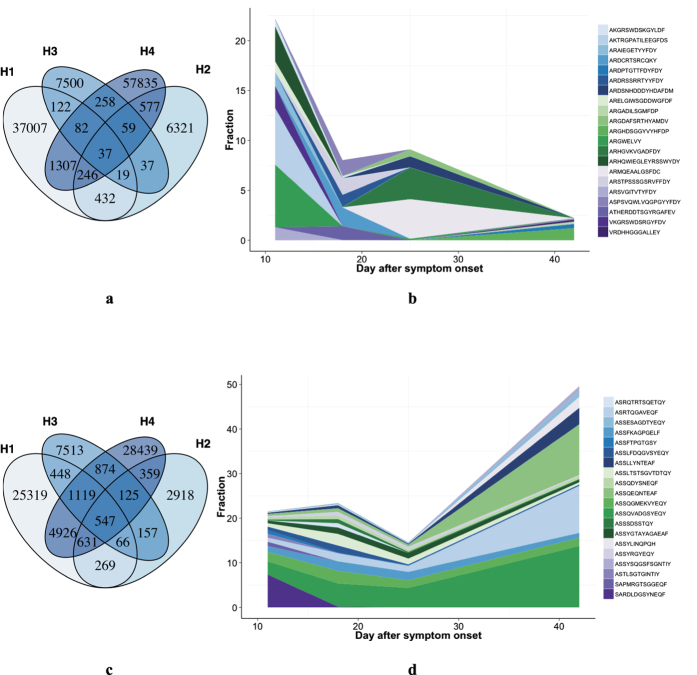
Dynamic change of IGH and TRB repertoires in an influenza A (H7N9) virus infected patient. (**a**,**c**) shows the overlap of IGH and TRB CDR3 sequences at different time points—11, 18, 25 and 42 days after symptom onset in patient H (survivor). (**b**,**d**) shows the dynamic changes of fractions of dominant IGH and TRB clones. Each CDR3 used a unique color. Width of each CDR3 clones stands for the fraction of this clone at each time point. CDR3 clones ranked top 5 in IGH repertories or top 20 in TRB were selected as representative dominant clones.

An important feature of the immune response to foreign pathogens is clonal expansion of specific T and B cells and their subsequent contraction. To track the longitudinal immune repertoire dynamics in response to H7N9, we tried to collect sequential samples from these patients. For one of these patients, a total of four samples at different time points (11, 18, 25, and 42 days, respectively) were collected, while three or less were collected from others. During the disease progression, IGH repertoire of patient H showed typical alterations that are also found in other patients. The IGH sequences exceptionally high variation between time points (Fig. 1a), which shared sequences consisted only 0.6 ~ 20% of the whole repertoire. In addition, the dominant clones of IGH repertoires changes overtime (Fig. 1b), indicating that the consistent part of the IGH repertoire was very limited. In contrast, the TRB repertoires presented a more stable pattern, with dominant clones conserved and constant part during the infection made up 23 ~ 61% of the whole repertoires (Fig. 1c,d). These results reveal the overwhelming variation as a property of IGH repertoire after H7N9 virus infection that differs from TRB repertoire.

### Diversity of Ig heavy chain repertoire increases and is positively related to patient prognosis

**Figure 2:**
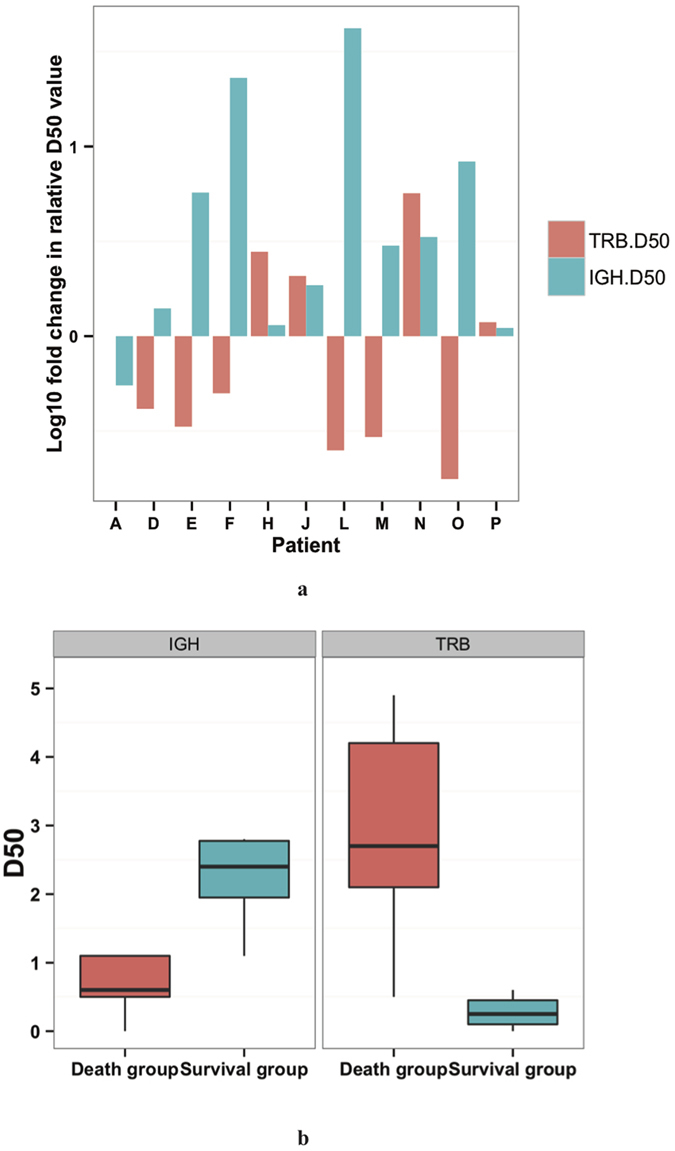
D50 values of IGH and TRB repertoires in H7N9 infected patients. (**a**) shows the logged relative ratios of IGH (green) and TRB (red) D50 values of samples collected from each patient. The D50 values >15 days were divided by D50 value <15 days and the logged result were drown as vertical axis values. Every pair of green and red bars represented an individual. (**b**) is the boxplot of the D50 value of IGH and TRB repertoires in 15–42 days after onset by group.

**Figure 3:**
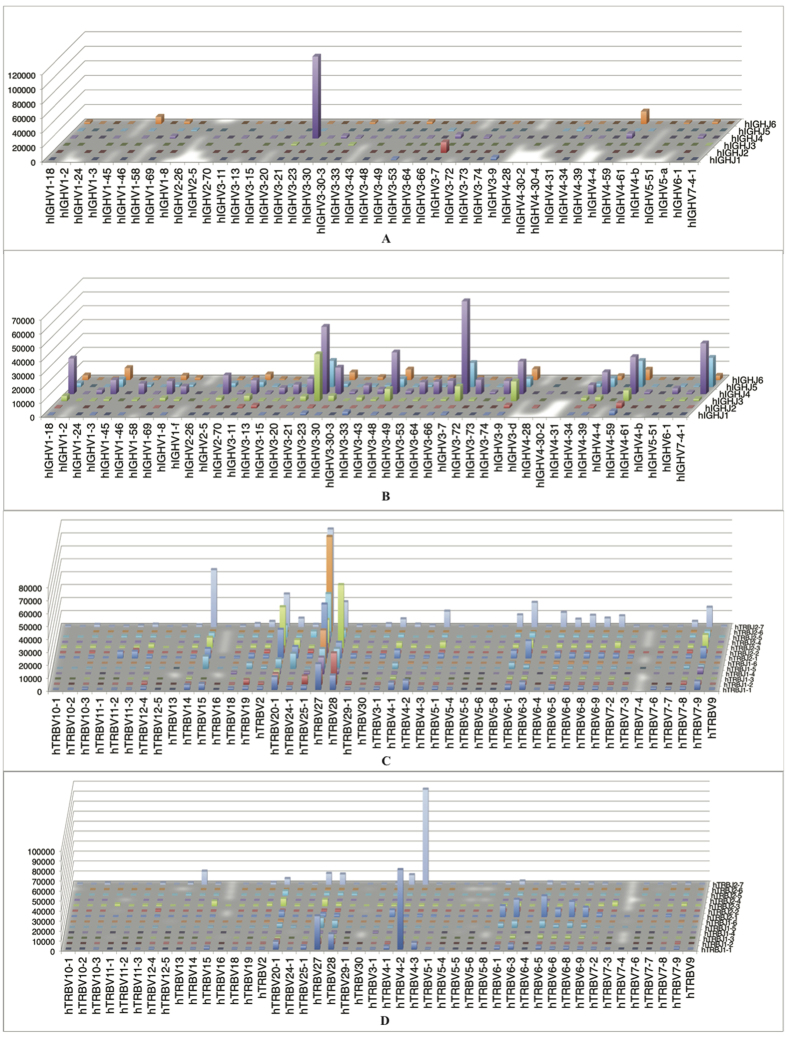
Comparison of overall diversity of V-J gene pairing in IGH and TRB repertoires between. (**A**,**B**) were representative IGH repertoires from a non-survivor and a survivor, respectively. (**C**,**D**) were TRB repertoires from the same patient as A and B at the same time point. Diversity of IGH repertoires was higher in survivor, while that of TRB repertoires was lower in survivor (Supplementary Figure S3).

Variation in immune response efficiency results in different infection severity and outcome. A proper diversity of the immune repertoire is crucial in order to mount an efficient adaptive immune response^15^. The overall D50 value of TRB and IGH in patients ranges from 0 ~ 5, indicating the repertoires are highly skewed (Fig. 2). For IGH repertoire, interestingly, the D50 values after 15 days were higher than the previous sample in all but one individual (Fig. 2a), suggesting an increasing tendency in IGH diversity. This is also the time point of neutralizing antibody (Nab) titers increasing in serologic measurements^16^ (Supplementary Figure S2). In contrast, the diversity of TRB repertoires did not increase over time. To simplify data analysis, we used the last sample of each patient, and the disease phase of these samples were comparable between groups (15–40 days in non-survival group and 15–46 days in survival group after onset). Importantly, D50 values of IGH repertoires were significantly lower in non-survivors than in survivors (P = 0.02, *Student’s t test)* (Fig. 2b), as were the Nab titers (Mann-Whitney U test, P = 0.04). On the contrary, survivors had lower D50 values of TRB repertoire than non-survivors (P = 0.02, *Student’s t test*) (Fig. 2b). As for IGL and IGK, the D50 values were comparable between the two groups (data not shown). For a more intuitive comparison of V-J rearrangement diversity of these repertoires, we performed 3D mapping of V-J pairing of two patients with different outcomes as examples (Fig. 3, Supplementary Figure S3). It is evident that the IGH repertoire of the non-survivor was significantly more convergent to several particular IGHV-IGHJ gene pairs than the survivor. On the contrary, the diversity of TRBV-TRBJ gene pairs in non-survivors was higher than that in the survivors. V-J pairs in IGK repertoire also skewed in the non-survivor (Supplementary Figure S3). These results, along with the finding that the IGH repertoire changed constantly over time in a single individual, indicate that as compared to other repertoires, the IGH repertoire diversity might be more associated with the patients’ recovery and the lower TCR diversity is related to the better outcome.

### Recombination patterns of V and J gene segments showed conserved patterns among patients

**Figure 4:**
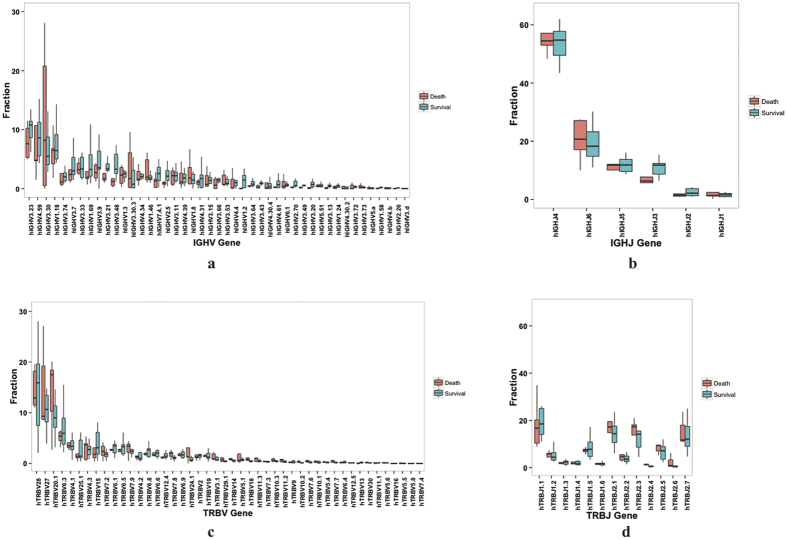
V and J gene usage in H7N9 infected patients by outcome. (**a**) shows frequency of IGHV gene usage in patients from non-survival group (red) and survival group (green). IGHV3-23 (9.73%), IGHV4-59 (8.76%) and IGHV3-30 (8.33%) were the three most frequent IGHV genes. (**b**) shows IGHJ gene usage frequency among patients. Expressing of IGHJ4 composed 44 ~ 64% of the repertoires. (**c**,**d**) show frequencies of TRBV and TRBJ genes usage. TRBV28 (14.28%), TRBV27 (12.69%) and TRBV20-1 (10.77%) accompanied by TRBJ1-1 (18.23%), TRBJ2-1 (16.31%) and TRBJ 2-3 (15.67%), were the three most frequent TRBV and TRBJ genes. Data are represented as mean ± SEM (See also Supplementary Figure S2).

To further investigate H7N9-specific alterations of immune repertoire during infection, we questioned if there was distinct V or J gene or V-J pairing bias in survivors. For IGH repertoires, most of the IGHV and IGHJ genes and pairs were comparable across different patients (Fig. 4a, Supplementary Figure S4a). There were some IGHV and IGHJ genes of low frequency identified more in non-survivors than in survivors (Fig. 4b). These signatures are similar with previously reported antibody repertoires after vaccination^17^. In IGH repertoires, the V and J gene usage distribution pattern was similar in each unique CDR3 and all CDR3 clones (r = 9.59, p < 0.001) (Supplementary Figure S4b). This suggests that the biased usage of V and J genes was not only a result of the high expression level of some clones but also the expansion of particular cell clones in IGH repertoire. Frequency of TRBV and TRBJ genes are also not significant between survivors and non-survivors (Fig. 4c,d), indicating that V-J gene usage is biased in patients but not related to their prognosis.

### Immune repertoires showed less CDR3 overlap in Ig repertoire across different individuals

Trimming and addition during combination effects the length of CDR3s, because the length of CDR3 loop influences its shape and ability to fold both on itself and in the company of others loops such as the CDR1 and CDR2^18^,^19^. We found that in some patients, amino acid lengths of unique IGH CDR3s using IGHV1-69 gene were skewed to a particular length, resulting in a significant perturbation of overall distribution (Supplementary Figure S5).

**Figure 5:**
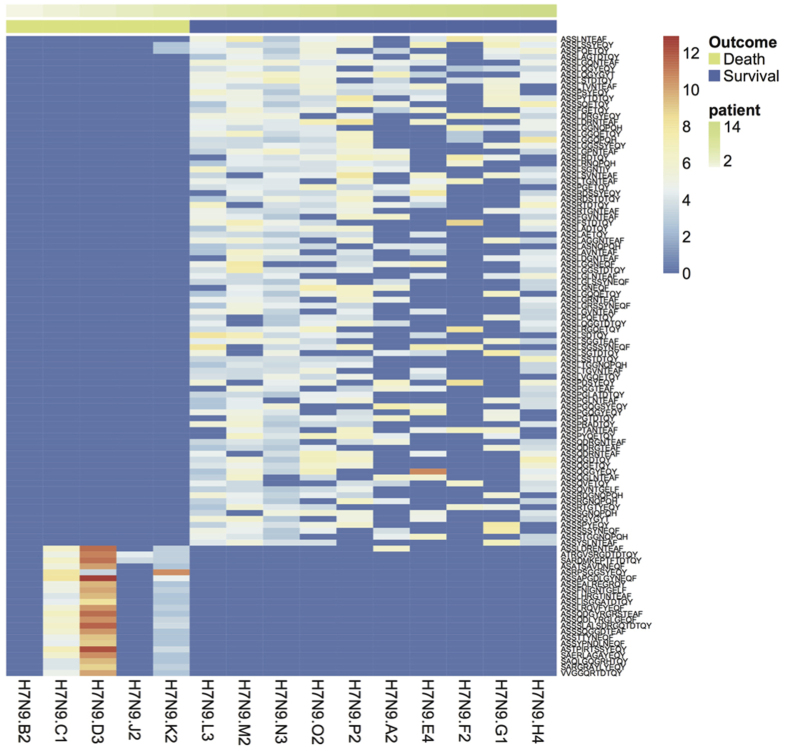
Shared TRB CDR3 sequences expressed at different levels between survivors and non-survivors. Comparison of the expression level of the shared sequences was performed using Wilcoxon Test, and the representative sequences expressed significantly different between survivors and non-survivors were drawn (p < 0.05). Each Colum is for one patient and the annotation bar represents the outcome of the patient (green for non-survival group, purple for survival group). Color of each rectangle stands for logged reads of the clone noted at right sided of the panel (blue as lowest, red as highest).

We compared the sequence identity of IGH CDR3s in response to H7N9 across different individuals. Overlap between different patients was very limited, even in intergroup pairs (data not shown). In addition, we failed in identifying any IGH sequences differently expressed in two groups. However, we found some TRB, IGL and IGK CDR3 sequences shared among most of the patients. Interestingly, survivors shared many TRB CDR3 sequences that found to be absent or expressed at a much lower level in non-survivors (p < 0.05) (Fig. 5). These shared sequences in TRB were predominately not dominant clones but of high similarity, which indicates that these sequences may have similar function and are related to recovery from infection. For IGK and IGL, patients with different prognosis expressed comparably high level of same or similar CDR3 sequences, including dominant clones (Supplementary Figure S6).

### Identifying broadly-neutralizing and antigen-specific antibody sequences from Ig repertoires

Although our results suggest that the diversity of IGH repertoire was associated with prognosis in H7N9 patients, it remains a significant challenge to understand the immunological basis of this observation. It has been reported that the human antibody repertoire becomes highly skewed in response to influenza vaccination with some B cell clones expanding in the setting of decreased diversity^20^, which may suggest that the immune system was primed to produce specific influenza-specific antibodies. However, we found that the diversity of IGH repertoire presented a rising trend during H7N9 infection, accompanied by the increase of Nab titer (Supplementary Figure S2). In addition to this, the survivors present the higher diversity and Nab titers compared to non-survivors, suggesting that the extensive B cell clonal expansion is not necessarily correlated with improved outcomes, and that human immune system follows a different pattern in response to natural infection as compared to vaccination.

**Figure 6:**
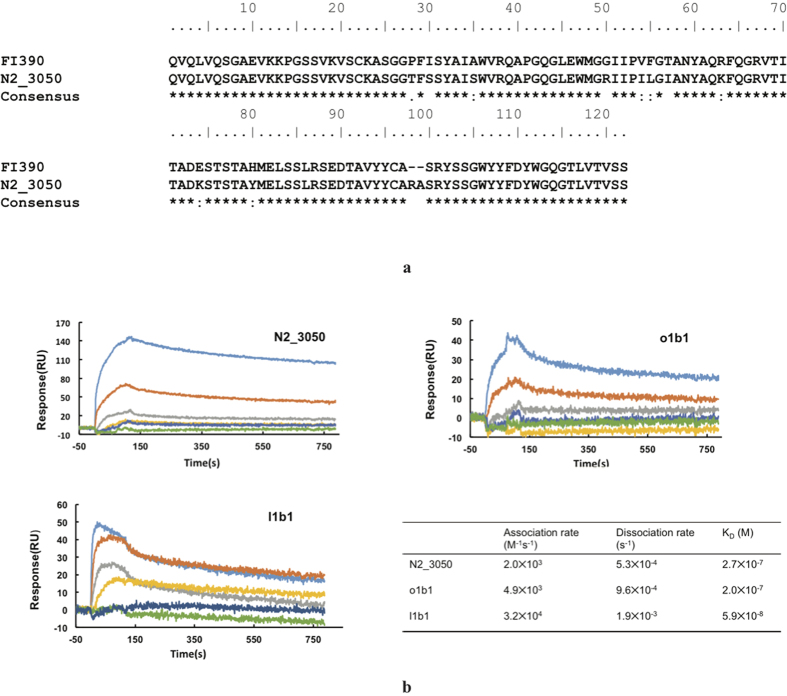
Identification of broadly neutralizing antibody (bnAb) and H7N9 specific antibody in Ig repertoires of H7N9 infected patients. (**a**) Alignment of previously reported bnAb sequence (FI390) and the antibody sequence found in H7N9 patient. FI390 was as a bnAb against influenza virus reported by Pappas L. N2_3050 was the IGH sequence found in two recovered H7N9 patients with identical CDR3 amino acid sequence as FI390. Alignment was performed using Clustal W2. (**b**) Binding kinetics of scFvs N2_3050, o1b1 and l1b1 to H7N9 HA antigen using Surface Plasmon Resonance assays. Different concentrations (10 uM, 2 uM, 400 nM, 200 nM, 100 nM and 50 nM for N2_3050 and c1b1; 1 uM, 500 nM, 250 nM, 125 nM, 62.5 nM and 31.5 nM for l1b1, respectively) is used in the test. Calculated K_D_ values are shown in the table presenting binding affinity of each scFvs.

To examine whether the highly diverse IGH repertoires in some H7N9 patients correlate with the presence of neutralizing antibodies, we firstly compared our deep sequencing data to the reported influenza bnAbs sequences. Surprisingly, we found a panel of clones that have almost identical IGH CDR3 with a previously reported pan-influenza A neutralizing antibody, FI390^21^. As shown in Fig. 6a, one of these clones, N2_3050, shared ~92% identity with FI390, with a difference of only seven residues in all three CDR regions. This convergence is remarkable considering that IGH repertoires are very diverse among different people, and not a single shared IGH CDR3 sequence can be found in the H7N9 patients investigated here. All these clones were from the samples of two survivors, collected during the convalescent phase (P2, N2). These results suggest that bnAbs may have been elicited in some patients.

We next sought to identify H7N9-specifc lineage members from IGH repertoires. We recently identified a human neutralizing antibody (Supplementary Table S5) from a non-immune human antibody library, which specifically neutralizes H7N9 influenza virus. We searched our deep sequencing database on the basis of in the last blood sample from all patients for sequences that could be clonally related to this antibody as they share the same IGHV1-69 and IGHJ1 germline families with a 14 amino acid long HCDR3. Interestingly, a number of clonally related sequences have been successfully identified in 8 of 10 H7N9 survivors, but have not been found in any of the non-surviving patients. Similarly to bnAb-related clones identified in patients P and N (survival group, 0.002–0.008% of the B-cell repertoires), these possible H7N9-specific lineage members represent a minor population (0.0001–0.01%) within the B-cell repertoire of each patient.

To further validate the functions of identified sequences *in vitro*, we synthesized some clonally related sequences, expressed the antibodies in scFv format, and measured their binding affinities to H7N9 HA (Table 1). 5 VHs that have similar sequences to our previous identified H7N9 antibody, 3 VHs that have similar sequences to another recently reported H7N9 human antibody^22^, as well as the VH of clone N2_3050 that shares 92% identity with the previously reported influenza A neutralizing antibody FI390, were fused with an identical VL to produce the scFv antibodies. We found that 5 of these 9 constructs could be solubly expressed in E. Coli, while the other 4 scFv only formed inclusion bodies. We next measured their binding to H7N9 HA using a SPR assay. As shown in Fig. 6b, we found that although scFvs e3b3 and m2a1 did not bind H7N9 HA, all the other three scFvs, N2_3050, o1b1 and l1b1, showed evident binding with different affinities (2.7 × 10^−7^, 2.0 × 10^−7^ and 5.9 × 10^−8^, respectively). The relatively low affinity is probably due to the fact that it was impossible to predict the naturally occurring VLs that were paired to VHs in these scFvs. Despite this, these results suggest that functional H7N9-specifc lineage members could be predicted from IGH repertoires.

Taken together, these results may suggest that a higher B-cell repertoire diversity in H7N9 infected patients is associated with the efficient production of neutralizing antibodies and, in turn, a better clinical outcomes.

## Discussion

New research has highlighted alterations in the T cell and B cell repertoires following vaccination and natural infection with the influenza virus^17^,^23^,^24^. Here we analyzed TRB and Ig repertoires of patients infected with influenza H7N9 virus in 2013, reported their diverse signatures, and examined their relationship with outcome. Furthermore, we report the development of antibodies specific to H7N9 and suggest that their presence may correlate with higher IGH diversity and favorable outcomes.

During acute viral infection, the immune system is activated with clone expansion, resulting in a more oligoclonal library^25^. In this study we used a novel index for describing and compare the diversity between immune repertoires—D50. In contrast to the previously reported Shannon index and Simpson index, the D50 value is understandable and direct. Meanwhile, relevance between these two indexes is impressive (Supplementary Table S3). Diversity of IGH was positively correlated with outcome. Effective somatic hyper-mutation and affinity maturation of B cells may be a possible mechanism for the high diversity and better outcome in survivors. Moreover, combined with the serologic studies, the IGH diversity showed increasing trend overtime accompanied with the increase of the Nab titer, indicating a more diverse IGH repertoire might be associated with the capacity for producing protective antibodies.

How TCR diversity impacts effective immunity remains unclear. Our results suggest that compared to Ig repertoire, which benefits patients by maintaining the diverse to generate antibodies with higher affinity, distinct correlation of T cell diversity and prognosis attributes to their difference in antiviral mechanisms. Without the somatic hyper-mutation process in B cells, T cell diversity depends on baseline diversity of T cell before infection and level of clonal expansion after antigen simulation. As TCR diversity is thought to be a prerequisite for immune system to recognize various foreign antigens, some earlier studies have suggested that the acquisition of cytotoxicity is independent of TCR usage and its diversity^26^,^27^. However, an early and robust H7N9-specific T cell response is critical for quick recovery from infection^8^. A recent study shows that, impairment of T cell proliferation and survival, not activation or effector function, results in prolonged virus infection in mice because of lacking sufficient antiviral T cells^28^. The relatively low T cell diversity of H7N9-infected survivors implies a more intense T cell expansion that contributes to virus elimination. This T cell response may be attenuated in the non-survivors. Thus, manipulating the expansion of T cells can be anticipated as a potential therapeutic intervention to benefit these patients^29^.

Identical TRB sequences between two individuals range from 1–10%^30^,^31^. The presence of these public sequences is suggestive to selective pressures of thymic selection and environmental exposure^30^. We noted that there are more shared CDR3 sequences were highly expressed in survivors, even though they were challenged by the same pathogen as non-survivors. This prognosis associated overlap suggests the effective TRB repertoire response is another possible mechanism for generating public CDR3s.

Importantly, our results provide evidence that functional neutralizing antibodies against the H7N9 virus could be identified using NGS methods. It has been recently recognized that different people may have convergent IGH gene rearrangements in their response to influenza vaccination^20^. In this study, we found that a number of sequences with stereotypical features of neutralizing antibodies can be identified from their B cell repertoires. We expect that the further improvement of deep sequencing related techniques, as well as a more comprehensive understanding of human antibodies, will enable a novel strategy to identify functional antibodies via immunogenetic analysis without the need of extensive *in vitro* screening procedures.

Notably, we have successfully identified a panel of sequences that could be clonally related to a previously reported bnAb. To the best of our knowledge, this is the first time that a bnAb against H7N9 virus has been identified in individuals without documented vaccination records. Recently, the discovery of bnAbs has renewed the interest in designing vaccines to elicit similar pan-influenza neutralizing antibodies *in vivo*^21^,^32^,^33^,^34^. However, the elicitation of these bnAbs has been found to be extremely challenging, because they only naturally arise in a small fraction of individuals after years of viral infection, and are highly divergent from their putative germline predecessors^21^. Therefore, a current priority is to understand how these antibodies have been elicited and mature *in vivo*, and translate this information into vaccine design. Interestingly, our results highlights the capability of deep sequencing techniques to identify and track the nAbs-related B cell clones *in vivo*, which will provide a foundation for understanding the mechanism of bnAb development and facilitate the development of possible “universal” influenza vaccines.

Our study has several limitations. Blood samples were taken from limited number of patients receiving standard treatment upon first pandemic of H7N9 virus. Some of them were treated with antibiotics, glucocorticoids, or anti-viral agents according to guidelines. However, the effects of these agents on immune repertoires have not been reported. Because H7N9 is a novel avian-origin influenza virus that had not been detected in humans previously, diagnosis in few patients was delayed, and thus immune repertoire changes at early stage of infection remain unclear. Despite of these, our results demonstrated a correlation between T cell and B cell diversity and patient outcome, suggesting a potential role of immune repertoire in influenza infection recovery.

This work represents the first time NGS has been used to analyze the immune repertoires of patients infected with the H7N9. The results reveal specific changes in human immune repertoires in response to H7N9 and provide insights into the immune repertoires that might provide improved antiviral immunity and thus improved recovery from infection. With further research in the field of immune repertoires, these results may have utility in assessing vaccine responses, and also in identifying antigen exposure or clinical diagnosis and evaluation, as well as antibody and vaccine development.

## Methods

### Patients

From March 2013 to June 2013, 18 individuals were diagnosed with H7N9 infection by viral isolation from throat swabs. Due to patient mortality and difficulty in providing follow-up, a total of 15 patients were enrolled in this study. Clinical characterization of these patients was presented in Supplementary Table S1 in the online data supplement, and management of individuals has been reported in our previous work^4^. All patients received anti-viral treatment. Among these patients, 5 died and the others survived to hospital discharge. The age, gender, and rate of complications are comparable between survivors and non-survivors. Blood samples were collected at multiple time points during critical illness. This study was approved by institution review board at Shanghai Public Health Clinical Center. All patient managements and blood sample collection were carried out in accordance with the relevant guidelines. Informed consents were obtained from all patients.

### Neutralizing antibody assays

We used the previously describedvpseundovirus with non-replicative human immunodeficiency virus backbone carrying influenza A H7 and H9 to measure the neutralizing antibody titers of patient serum^35^. The highest serum dilution that gave ≥80% inhibitory concentration of the luciferase signal in virus-infected MDCK cells was defined as neutralizing titer.

### Isolation of T cells and B cells and extraction of RNA

From anticoagulated whole blood, peripheral blood mononuclear cells (PMBCs) were obtained by Ficoll-gradient according to the manufacturers’ instruction, and the isolated cells were resuspended in RNA protect reagent (Qiagen).

### Establishment of immune repertoires

We performed arm-PCR technology to amplify the cDNA semi-quantitatively according to the manufactures’ instructions (iRepertoire, Inc.) as has been described elsewhere^36^. Briefly, in the first few cycles, we used nested primers (Fo, Fi, Ro, Ri), which were designed specifically for every V and J (or C) genes and shared a common tag sequence. After that, the nested primers were removed from PCR products by exonuclease, and a pair of communal primers, called “superprimer”, was added to pair with the common tag sequence in the products and accomplish the exponent phase. Unique barcoded primers were used to distinguish each sample. After gel purification, the library constructed by previous steps was pooled and underwent high-throughput sequencing based on Illumina MiSeq platform according to the manufactures’ protocol.

### Alignment of CDR3 sequences

Complimentary determining regions (CDR3s) were identified as the interval between two conserved amino acid sequences—Y[YFLI]C at the 3′ end of the V gene segment and [FW]GXGT (X stands for 1 of 20 amino acids) within the J segments. Raw data was analyzed by iRepertoire using the previously described IRmap program^36^. The best matches of germline V and J gene were searched by determining alignments between Illumina platform product and germline sequences in the IMGT/GENE-DB database.

### D50 value for diversity comparison

To make the diversity comparison easier and to analyze diversity statistically, we used a new index – D50 value in our work. It is the calculated percentage of dominant unique clones, accumulative reads of which made up for 50% of the total (ranges from 0 to 50 in theory) (Supplementary Table S2, Supplementary Figure S1). To stress clone expansion, avoid the bias from sequencing depth and reduce noise, we used the sequences ranked within 10000 when calculating and excluded others. D50 value of a specific repertoire is positively related to diversity.

### Antibody expression

The scFv gene were synthesized by Genscript (Piscataway, NJ) and cloned into pComb3x vector. The plasmids were transferred into HB2151 cells, and freshly single colonies were inoculated into SB medium and induced by 1 mM isopropyl-1-thio-β-D-galactopyranoside for large-scale expression. The antibodies were purified by nickel-nitrilotriacetic acid resin (Qiagen, Valencia, CA) according to the manufacturer’s protocols. Protein purity was estimated as >90% by SDS–polyacrylamide gel electrophoresis, and protein concentration was measured spectrophotometrically (NanoVue, GE Healthcare).

### Surface Plasmon Resonance binding experiments

The binding experiments were performed using a ProteOn XRP36 system (Bio-Rad, Hercules, CA) to determine the kinetics of scFvs N2_3050, o1b1 and l1b1 to H7N9 HA antigen (Sino Biological Inc.). H7N9 HA was immobilized on the ProteOn GLM biosensor chip using standard amine coupling chemistry (300 nM in 10 mM sodium acetate buffer, pH 5.0). The surface of sensor chip was activated by 200 mM 1-ethyl-3-dimethyl aminopropylcarbodiimide hydrochloride and 50 mM N-hydroxysulfosuccinimide. O1b1 and l1b1 were prepared in phosphate buffer saline (pH 7.4) containing 0.005% Tween-20 and injected at 50 μl/min for 120 s at different concentrations (10 uM,2 uM, 400 nM, 200 nM, 100 nM and 50 nM for N2_3050 and c1b1; 1 uM, 500 nM, 250 nM, 125 nM, 62.5 nM and 31.5 nM for l1b1, respectively). The dissociation phase was followed for 600 s. The chip surfaces were regenerated by injecting 10 mM glycine HCl, pH 2.0, 100 μl/min for 18 s. The data were analyzed using ProteOn Manager 3.1 software and fitted to a 1:1 interaction model.

### Data analysis

When comparing the reads between samples, we used fraction or normalized the reads to a total of 10,000,000 to avoid any bias caused by sample size. The comparison of D50 index was performed using Student’s t test. Expression level of each sequence or V-D-J gene and was compared by Wilcoxon test or Mann-Whitney U test, respectively. Statistical analysis was conducted using SPSS software (version 21.0) and R (3.1.2). Structured Query Language (SQL) was used for immunogenetic analysis. Sequence alignments were made with ClustalW2.

## Additional Information

**How to cite this article**: Hou, D. *et al*. Immune Repertoire Diversity Correlated with Mortality in Avian Influenza A (H7N9) Virus Infected Patients. *Sci. Rep.* **6**, 33843; doi: 10.1038/srep33843 (2016).

## Supplementary Material

Supplementary Information

## Figures and Tables

**Table 1:** Expression and HA7 binding affinity of sequences clonally related to H7N9 antibodies.

Sequence ID	V gene	D gene	J gene	CDR3	Expression	Function
N2_3050	IGHV1-69*09	IGHD6-19*01	IGHJ4*02	ARASRYSSGWYYFDY	Yes	Yes†
o1b1	IGHV1-69*01	IGHD1-20*01	IGHJ3*02	ARSNYNPLLAAFDI	Yes	Yes
l1b1	IGHV1-69*01	IGHD5-18*01	IGHJ5*02	ARGYSYGLREWFDP	Yes	Yes
e3b1	IGHV1-69*09	IGHD7-27*01	IGHJ4*02	ARDAGSTWGIYFDS	No*	No
e3b3	IGHV1-69*02	IGHD2-21*02	IGHJ3*01	ARDPAGGDRDAFDV	Yes	No
c1b1	IGHV1-69*09	IGHD4-17*01	IGHJ5*02	ARGLNYGDVGWFDP	No	No
m2a1	IGHV4-31*03	IGHD3-3*01	IGHJ6*03	ARDGPYYDFWSGREPDV	Yes	No
p2a1	IGHV4-31*03	IGHD4-17*01	IGHJ6*02	ARANYGDRHTYYYGMDV	No	No
p2a3	IGHV4-31*03	IGHD4-17*01	IGHJ6*02	ARGDYGDRYYYYYGMDV	No	No

IGH- immunoglobulin heavy chain; TRB – T cell receptor beta chain.

^*^No Expression means scFv only formed inclusion bodies.

^†^Antibodies with KD(M) < 10^−7^ are considered to be functional.
