# Supplementary material for: Immune Repertoire Diversity Correlated with Mortality in Avian Influenza A (H7N9) Virus Infected Patients
Source: Sci Rep. 2016 Sep 27;6:33843. doi: 10.1038/srep33843 (PMC5037391; doi:10.1038/srep33843)
Supplement: Supplementary Information [file srep33843-s1.pdf]

Immune Repertoire Diversity Correlated with Mortality in Avian Influenza A (H7N9) Virus Infected Patients

[1]Dongni Hou*, [2]Tianlei Ying*, [2]Lili Wang*, [3]Shuihua Lu*, [4]Eric Seeley*, [3]Jianqing Xu*, [1]Qin Wang*, [1]Cuicui Chen*, [3]Xiuhong Xi, [3]Tao Li, [1]Jie Liu, [1]Xinjun Tang, [3]Zhiyong Zhang, [1]Jian Zhou, [1]Chunxue Bai, [5]Chunlin Wang, [5]Miranda Byrne-Steele， [6]Jieming Qu[#]， [5]Jian Han[#], [1,3,7]Yuanlin Song[#]

Online Data

Supplementary Tables and Figures

Table S1 Clinical Figures of 15 Patients Infected With H7N9 Virus

	Survivors	Non-survivors	P value
No. (%)	10(66.6%)	5(33.3%)	
Age (median)	68.5(47-81)	80(56-88)	0.538*
Gender (Female)	2(20%)	1(20%)	1.000†
Underlying disease[a]	8(80%)	3(60%)	1.000†
Complications			
ARDS[b]	0(0%)	5(100%)	0.000†
Severe pneumonia	1(10%)	4(80%)	0.017†
Bacterial infection	2(20%)	3(60%)	0.251†
Treatment			
On Tamiflu	10(100%)	5(100%)	1.000†
Corticosteroids	6(60%)	5(100%)	0.231†
Antibiotics	10(100%)	5(100%)	1.000†

[a] Underlying disease included hypertension, diabetes, cardiovascular disease, liver disease, rheumatoid arthritis, as well as chronic obstructive pulmonary disease. [b]ARDS stands for acute respiratory distress syndrome. [c] Time between onset of symptoms and death or discharge.

*P value of Student's t test. †P value of Fisher exact test. Continuous variables were summarized as means with ranges.

			Simpson	Shannon	D50
Spearman rho	Simpson	Correlation index	1.000	.689**	.808**
		Sig. (bilateral)	.	.004	.000
		N	15	15	15
	Shannon	Correlation index	.689**	1.000	.542*
		Sig. (bilateral）	.004	.	.037
		N	15	15	15
	D50	Correlation index	.808**	.542*	1.000
		Sig. (bilateral)	.000	.037	.
		N	15	15	15

**. Spearman correlation index P<0.01 *. Spearman correlation index P<0.05

Table S2 Spearman correlation between Simpson index, Shannon index and D50

sample ID	out-come	sample time	TRB				IGH				IGL				IGK			
			Rea	CDR3	%	Uniq	Reads	CDR3	%	Uniq	Read	CDR	%	Uniq	Read	CDR3	%	Unique
**A1**	S	15	838	832490	99	5426	199436	196006	98	15146	1357	1344	99%	472	63742	62323	98%	4800
**A2**	S	22	932	927617	99	11444	527986	519458	98	32423	1128	1108	98%	557	21735	212113	98%	7181
**B2**	D	16	337	333140	99	914	101761	100923	99	4655	132	131	99%	101	26564	264507	100	2305
**C1**	D	15	350	340552	97	3099	57292	56430	98	3132								
**D1**	D	10	568	561143	99	19671	177282	176288	99	45547	150	143	95%	77	97193	967569	100	10604
**D2**	D	17	460	450895	98	4423	517855	512968	99	24716	1704	1642	96%	315	43404	422085	97%	8808
**D3**	D	24	643	635673	99	7996	778236	773330	99	19895	12	12	100%	10	70583	700969	99%	6426
**E2**	S	15	727	713141	98	7991	292663	290733	99	11242	622	618	99%	162	41356	408960	99%	7449
**E3**	S	22	589	582542	99	15902	817274	810486	99	40371	80	80	100%	72	68907	682642	99%	15037
**E4**	S	29	721	713028	99	13474	333530	331140	99	18761	18524	18292	99%	1635	24929	246851	99%	6555
**F1**	S	13	113	112813	99	43581	114255	113481	99	35818								
**F2**	S	20	116	112590	97	4949	51562	50606	98	4307	315	301	96%	107	5814	5646	97%	967
**G1**	S	13	474	470371	99	6934	392082	387616	99	24640	132	131	99%	101	14207	139625	98%	4896
**H1**	S	11	795	787954	99	33326	935038	929827	99	39253	35683	35412	99%	1896	38251	380619	100	7591
**H2**	S	18	428	418663	98	5073	66233	65438	99	7729	1513	1445	96%	360	37447	36517	98%	3314
**H3**	S	25	116	114647	98	10850	221444	218673	99	8115	33490	32878	98%	4491	27455	270110	98%	14459
**H4**	S	42	685	679584	99	37021	822487	817033	99	60402	33591	32259	96%	1927	51739	513551	99%	16603
**J1**	D	8	368	352926	96	2025	242378	237098	98	12455	1185	1141	96%	250	17232	16881	98%	1469
**J2**	D	15	558	548936	98	2674	33472	32937	98	3146	24589	23839	97%	1699	35873	349393	97%	9786
**K1**	D	26	935	930298	99	55191	602394	598845	99	17388	12710	12451	98%	760	22472	222553	99%	4088
**K2**	D	40	128	127482	99	13024	225424	224247	99	4423	12167	12092	99%	1087	41330	411273	100	5291
**L1**	S	8	606	598756	99	43581	606705	603103	99	35398	2392	2329	97%	813	91271	903421	99%	16914
**L2**	S	15	122	118413	97	9623	451381	445206	99	13505	6747	6611	98%	815	31317	310052	99%	7008
**L3**	S	28	131	130299	99	69270	433485	430337	99	41484	17130	16917	99%	2687	32865	324890	99%	14609
**M1**	S	13	759	751677	99	7416	226530	224195	99	19175	12969	12506	96%	1977	24317	238526	98%	8130
**M2**	S	33	533	527876	99	48426	940612	931701	99	81567	12682	12418	98%	2584	43778	433876	99%	12630
**N1**	S	11	123	123028	99	60184	122070	121225	99	57612	78943	77145	98%	3399	10081	986242	98%	14733
**N2**	S	18	846	832718	98	4659	224162	215954	96	20996	2384	2126	89%	510	56153	51592	92%	3540
**N3**	S	46	112	111319	99	72136	302822	298423	99	60992	114	107	94%	90	2388	2321	97%	929
**O1**	S	15	742	711559	96	4304	195445	193925	99	7746	12	12	100%	10	21120	209664	99%	3788
**O2**	S	32	578	572201	99	49275	665024	659720	99	49253	37686	36732	97%	3666	70444	696065	99%	19345
**P1**	S	8	573	553956	97	11667	575911	569899	99	32274	19885	19726	99%	1223	77178	761758	99%	13520
**P2**	S	15	683	663861	97	26526	331662	328480	99	28727	22152	21947	99%	2415	74675	735776	99%	13605

*TRB—T cell receptor beta chain; IGH—immunoglobulin heavy chain; IGL—immunoglobulin lambda chain; IGK—immunoglobulin kappa chain

Table S3 High-throughput Sequencing Results of T cell and B cell Receptors of H7N9 Infected Patients

IGHV gene	IGHJ gene	HCDR1	HCDR2	HCDR3
**IGHV1-69**	IGHJ1	**GGTFSSYA**	**IIPIFGTA**	**ARDPSFWAAEYFQH**

Table S4 Sequence of an H7N9-Specific Neutralizing Antibody from a Non-immune Human Antibody Library

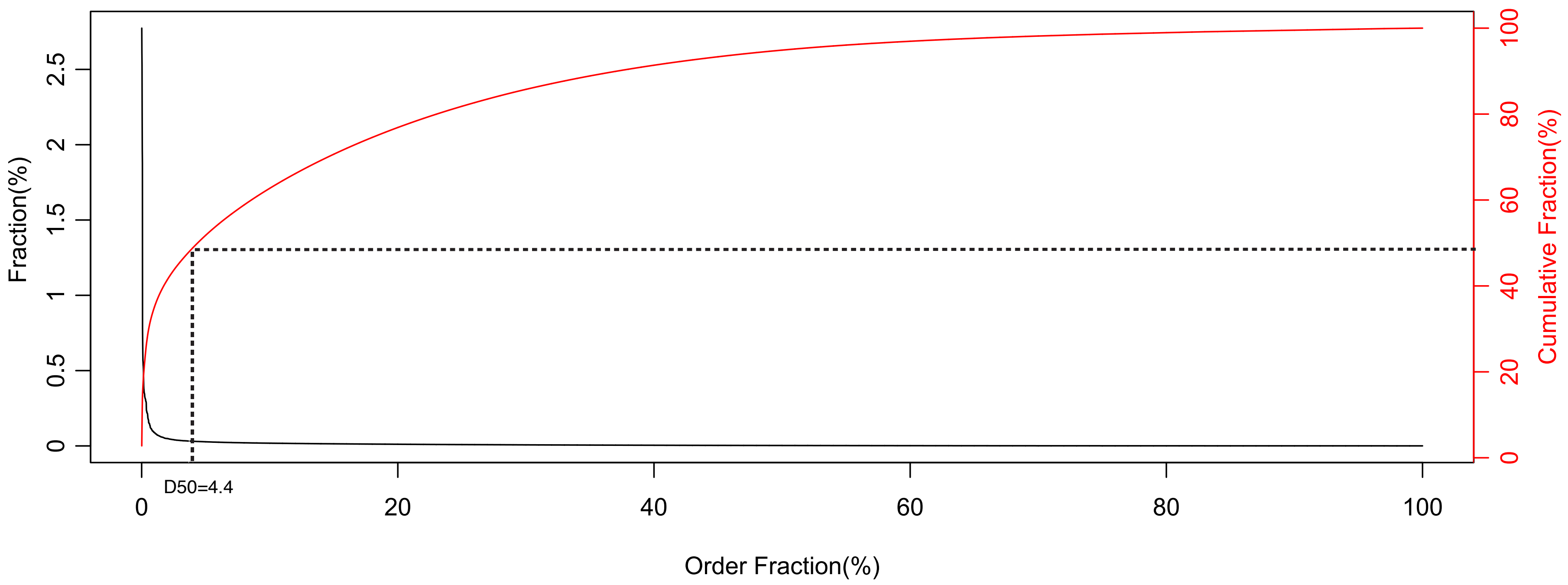

Figure S1 A graphical display of D50 value for diversity measurement

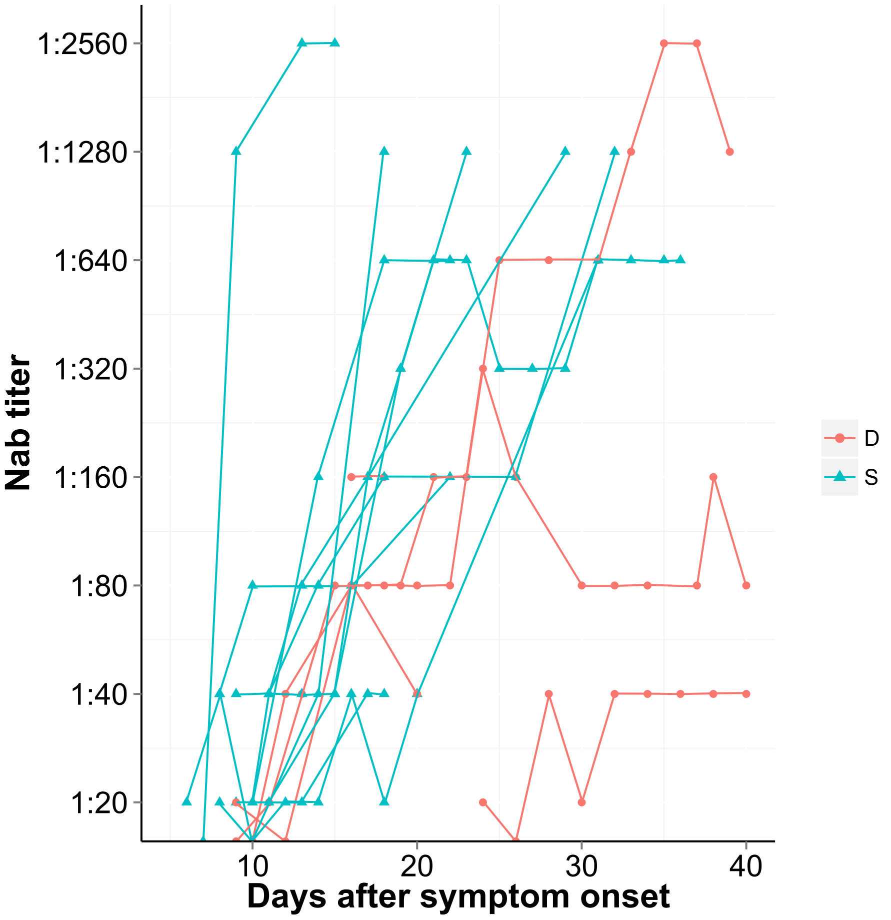

Figure S2 Neutralizing antibody (Nab) titers of influenza A (H7N9) patients after illness onset.

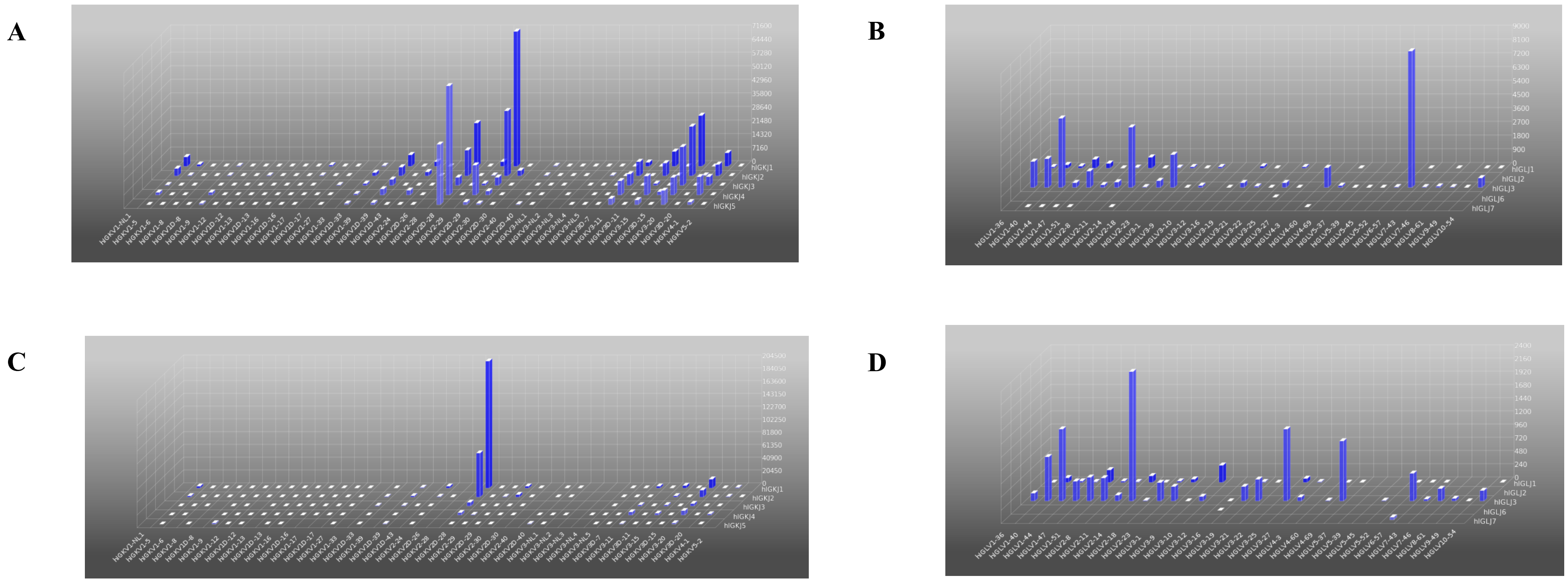

Figure S3 3D Map of IGL and IGK Repertoire of H7N9 infected patients. A and C were presentative IGK repertoires from a deceased patient (40d after symptom onset) and a survived patient (40d after symptom onset), respectively. B and D were IGL repertoires from the same patient as A and B at the same time point. Diversity of IGK repertoires was higher in the survivor, while that of IGL repertoires showed no significant difference. The two patients are the same with the patients in **Figure 3.**

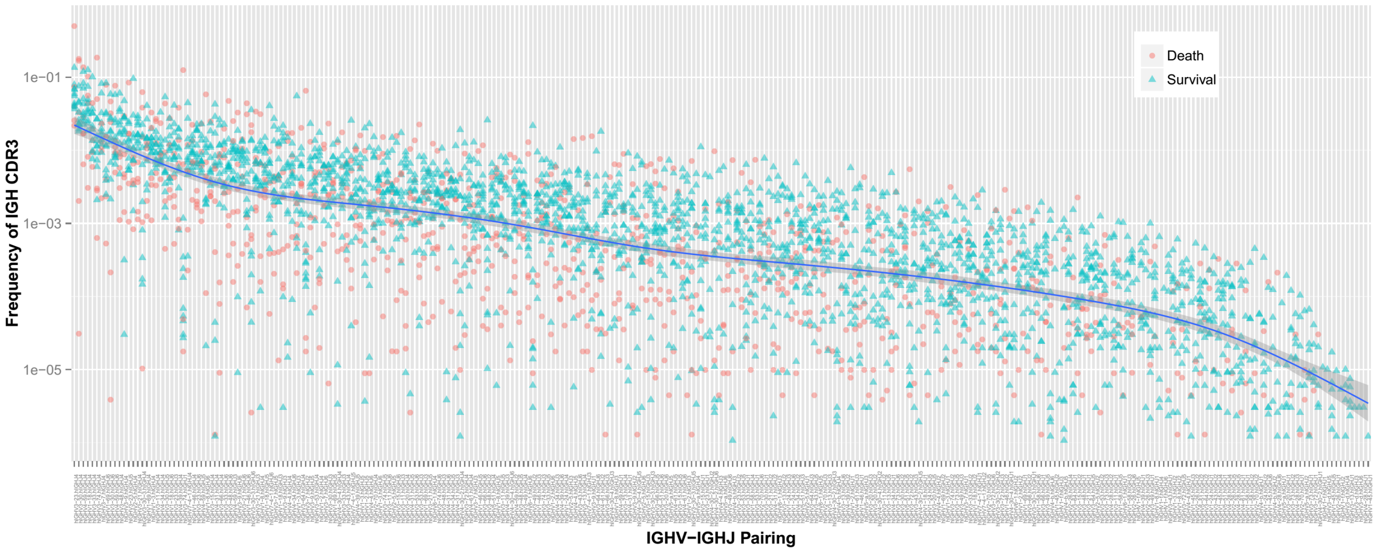

A

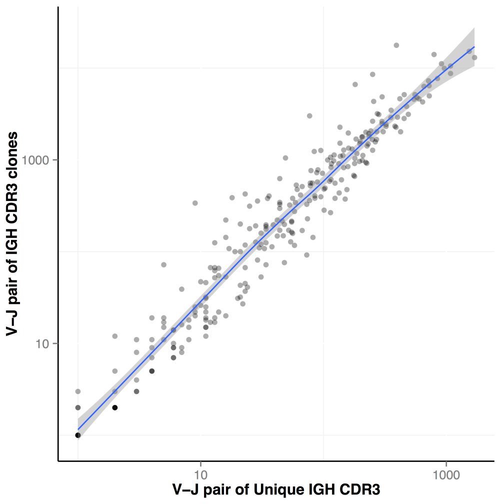

B **Figure S4 V-J pairing of IGH Repertoire in H7N9 Patients. A** shows the frequency of each V-J pair of IGH in patients infected with H7N9. Each column contains two points, which were the average percentages of non-survivor group and survivor group, respectively (red for non-survivors and blue for survivors). **B** shows the correlation of frequency of IGH V-J paring between different calculating strategies using all CDR3 sequences or the unique CDR3s (r=9.59, p<0.001).

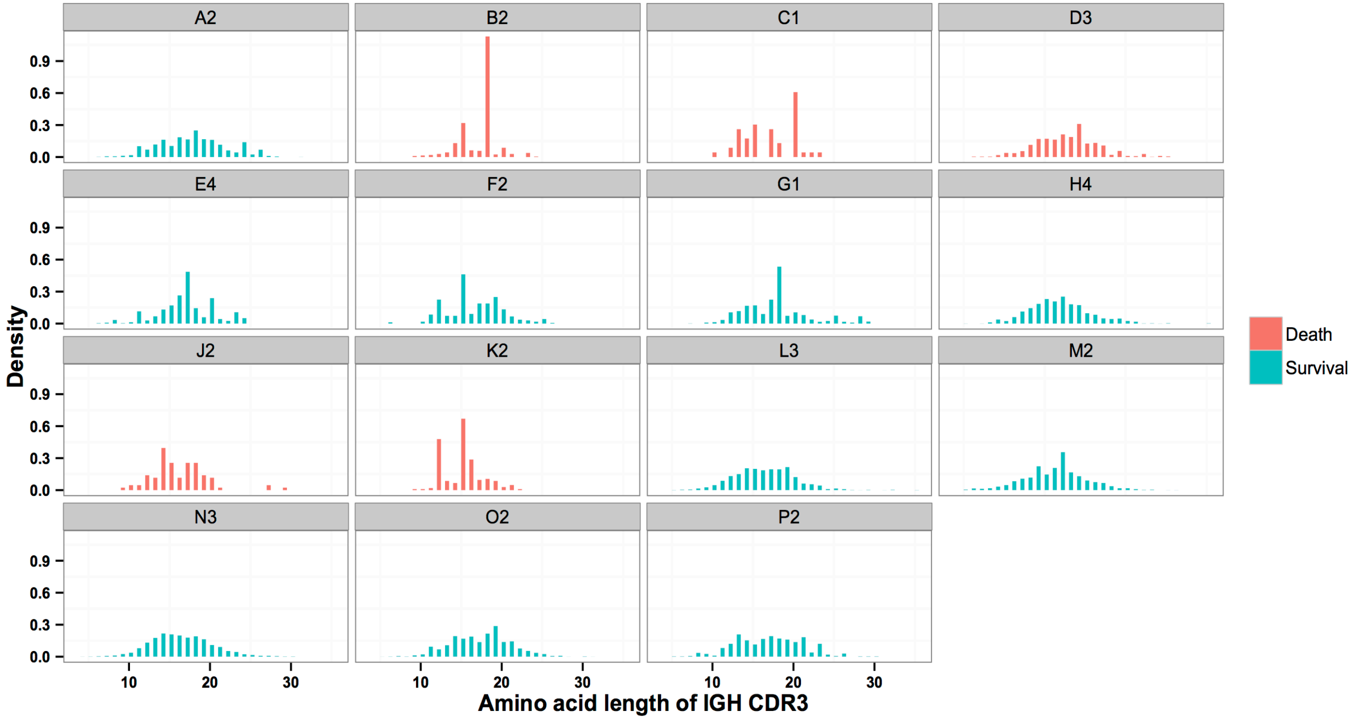

Figure S5 Distribution of Amino Acid Length of IGH CDR3 Sequences in IGHV 1-69 Gene Family. Histograms were colored according to patient outcome (red for non-survivors, green for survivors).

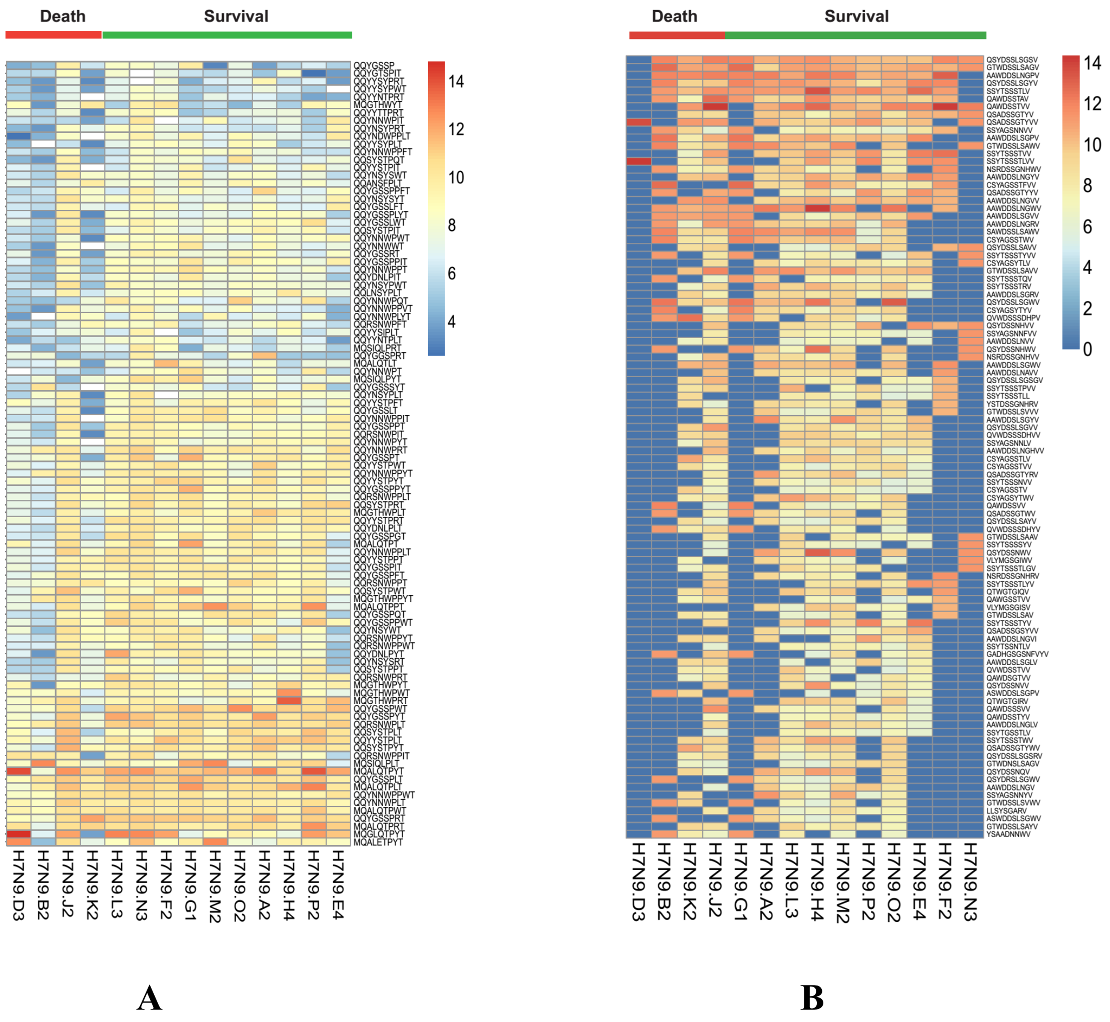

Figure S6 Shared IGL and IGK CDR3 Sequences among H7N9 Infected Patients by Outcome. The most prevalent IGL (A) and IGK (B) CDR3 sequences were selected as presentative sequences by row. Each Colum is for one patient and the annotation bar represents the outcome of the patient (green for non-survivor group, purple for survivor group). Color of each rectangle stands for logged reads of the clone noted at right sided of the panel (blue as lowest, red as highest).
